# *DSP1* regulates planarian (*Dugesia japonica*) neural regeneration depending on *CnB*

**DOI:** 10.3389/fcell.2026.1932725

**Published:** 2026-10-08

**Authors:** Lili Gao, Mingyue Zheng, Ziyuzhu Kong, Yutong Li, Jiaxin Li, Fengtang Yang, Zhonghong Cao, Hui Zhen

**Affiliations:** 1 School of Life Sciences and Medicine, Shandong University of Technology, Zibo, China; 2 School of Medical Laboratory, Qilu Medical University, Zibo, China

**Keywords:** CnB, DSP1, Dugesia japonica, neural regeneration, regulate

## Abstract

Regeneration and repair of the injured central nervous system is a major focus in biological research, yet many key questions remain unanswered, making it essential to explore the mechanisms underlying neural regeneration. Dorsal switch protein 1 (DSP1) plays critical roles in embryonic development and immune regulation in multiple invertebrates including *Drosophila*, whereas its vertebrate ortholog HMGB1 participates in diverse biological processes; however, its function in central nervous system regeneration has not been reported. In this study, we found that *DSP1* was widely expressed in the intact planarian, predominantly in parenchyma, retropharyngeal and cephalic ganglia. Knockdown of *DSP1* promoted blastema growth at one dpa, without affecting the stem cell population. Silencing of *DSP1* accelerated brain regeneration and significantly increased the number of daughter cells derived from stem cells, accompanied by downregulated expression of calcineurin subunit B (*CnB*), a Wnt signaling pathway gene. Notably, simultaneous knockdown of *DSP1* and *CnB* promoted blastema regeneration. These findings suggest *DSP1* is associated with *CnB* to regulate neural regeneration in planarian. These findings provide a theoretical basis for understanding the DSP1/CnB signaling axis in regulating neural regeneration in mammals, and have important implications for the development of drugs targeting neuronal regeneration.

## Introduction

Adult stem cells possess immeasurable medical value in the field of regenerative medicine, providing new strategies for the effective treatment of many major diseases. Neural stem cells serve as the cellular basis for neural self-repair and regeneration. Upon neural injury or neurodegenerative diseases such as Parkinson’s disease and Alzheimer’s disease, the body can rely on endogenous stem cells to repair damaged tissues and generate new neurons and synapses (Heneka et al., 2015; Li and Clevers, 2010). Accumulating evidence has demonstrated that endogenous neural stem cells confer regenerative repair capacity within the mammalian central nervous system (CNS) (Yao X et al., 2025; Naffaa, 2025). However, due to constraints imposed by the intrinsic properties of stem cells and the internal microenvironment, their differentiation capacity is limited, resulting in extremely restricted regeneration in the adult mammalian CNS (Taupin, 2011; Pilz et al., 2018). Investigating the processes and molecular mechanisms by which adult stem cells participate in neural regeneration *in vivo* may represent an important direction for the future development of regenerative medicine. Therefore, understanding the mechanisms by which endogenous stem cells regulate neural regeneration is of great significance for neural regenerative medicine.

Based on extraordinary regenerative capacity, planarians hold key evolutionary significance and serve as an excellent model for studying tissue regeneration and stem cell regulation. Upon amputation, each fragment can regenerate into a complete individual within approximately 1 week, and this robust regenerative ability is mainly attributed to the abundant population of stem cells (neoblasts), which account for about 25%–30% of the total cells (Zeng et al., 2018). Studies have demonstrated that neoblasts, as the only proliferative cell population in planarians, are classified into 12 subpopulations; among them, Nb2 represents a pluripotent stem cell population capable of generating all planarian somatic lineages, while Nb11 is a neural stem/progenitor cell population capable of proliferating and differentiating into the nervous system (Zeng et al., 2018). When planarians are injured, neoblasts are rapidly activated, enabling efficient regeneration of missing tissues and organs, including the CNS. The planarian CNS consists of a pair of inverted U-shaped cephalic ganglia (brain), photoreceptors (eyes), and ventral nerve cords extending along the body length (Cebria, 2007). Research has indicated that the planarian brain comprises approximately 2,000–10,000 neurons, including classical neuronal types conserved in humans, such as dopaminergic, cholinergic, and GABAergic neurons, and these neuronal subtypes are precisely restored in terms of spatial location and number after each regeneration event (Nishimura et al., 2007a; Nishimura et al., 2007b; Nishimura et al., 2010; Nishimura et al., 2008). Therefore, planarians can serve as an excellent animal model for studying neural regeneration and stem cell biology.

Studies have demonstrated that the proliferation, migration, and directed differentiation of stem cells during planarian neural regeneration are tightly regulated by a complex network of genes and signaling pathways. For instance, the Wnt signaling pathway mediates the establishment of the anterior-posterior axis in planarians, and downregulated expression of genes within this pathway can lead to posterior expansion of the brain (Kobayashi et al., 2007). The EGFR signaling pathway plays a crucial role in cell differentiation during planarian regeneration, with involvement in the early differentiation of neurons during head regeneration (Fraguas et al., 2014). Additionally, downregulation of *Tec-1* expression results in an increased in several neuronal subtypes during both planarian regeneration and tissue homeostasis maintenance (Karge et al., 2020). As a broadly expressed regulatory factor in planarian neural precursor cells, *Coe* participates in the generation of dopaminergic, cholinergic, and GABAergic neurons throughout neural regeneration (Cowles et al., 2014; Cowles et al., 2013). Our previous studies have demonstrated that the Wnt/Ca^2+^ signaling pathway regulated the formation of GABAergic neurons during planarian brain regeneration; downregulation of genes associated with this pathway resulted in multiple regenerative abnormalities, such as hypoplastic regenerated brain nerves, failure of lateral nerve regeneration, and a decrease in GABAergic neurons—effects primarily mediated by the regulation of neural cell differentiation (Zhen et al., 2020). However, the molecular mechanisms underlying the directed differentiation of pluripotent stem cells into neural stem cells in planarians, as well as the processes by which neural stem cells further differentiate into neurons and glial cells remain partly elucidated.

DSP1 is a homolog of vertebrate HMGB1 (high mobility group protein 1), which is widely expressed in the nucleus and regulates transcription and chromatin reorganization via DNA binding (Bianchi et al., 2017). Studies have demonstrated that DSP1 can be passively released from dying cells or actively secreted by activated immune cells, intestinal cells, hepatocytes, and other cell populations (Tsung et al., 2007); as a damage-associated molecular pattern (DAMP) molecule, it initiates the innate immune response (Roh and Sohn). *Aedes aegypti*, DSP1 facilitates chromatin remodeling of Toll-associated transcription factors, thereby enhancing their binding to promoters in response to immune challenges (de Mendonca Amarante et al., 2017). Upon bacterial attack in *Spodoptera litura*, DSP1 is released into the hemolymph and mediates various immune responses through PLA2 activation (Mollah et al., 2021). *In Drosophila melanogaster*, nuclear DSP1 functions as a co-activator of the Dorsal protein to modulate fly growth and development: it is ubiquitously distributed at the early stages of embryogenesis (cell blastoderm and germ band), restricted to the central nervous system at late embryogenic stages, and in adult *D. melanogaster*, DSP1 protein is exclusively detected in the ovaries and brain (Mosrin-Huaman et al.). Beyond insect models, functional studies of HMGB1 orthologs have also been conducted in other lophotrochozoan flatworms. In the trematode *Schistosoma mansoni*, a conserved HMGB1 homolog is expressed throughout larval and adult stages, participating in host immune modulation and tissue remodeling during parasitic infection (Gnanasekar et al., 2006). Collectively, these invertebrate studies demonstrate that DSP1 exerts core functions in embryonic development and innate immune regulation across protostomes, while its vertebrate ortholog HMGB1 mediates parallel immune and developmental programs in mammals (Mollah et al., 2021; Mosrin-Huaman et al.; Janke et al., 2003). However, whether DSP1 contributes to central nervous system regeneration remains unreported to date.

In this study, we found that *DSP1* was widely expressed in the intact planarian, predominantly in parenchyma, retropharyngeal (referring to the mesenchymal parenchyma posterior to the pharyngeal pouch) and cephalic ganglia. Knockdown of *DSP1* promoted blastema growth at 1 day post regeneration, without affecting the stem cell population. *DSP1* silencing accelerated brain regeneration and increased the number of daughter cells derived from stem cell differentiation, accompanied by downregulated expression of *CnB*, a Wnt signaling pathway gene. Notably, simultaneous knockdown of *DSP1* and *CnB* promoted blastema regeneration. These findings suggest a genetic interaction between *DSP1* and *CnB* in the regulation of planarian neural regeneration.

## Materials and methods

### Planarians

*Dugesia japonica* planarians were reared under controlled laboratory conditions: they were housed in spring water with a stable ambient temperature set at 20 °C. To eliminate potential interference from recent feeding, all planarians were subjected to a mandatory fasting period of no less than 7 days before the start of any experimental procedures. For the regeneration experiments, individual planarians (8–10 mm) were transected to remove heads; the resultant trunk fragments were then collected and used for a series of analyses at predetermined time points (0, 1, 3, 5, 7, and 10 days post-amputation (dpa)). For each time point (0, 1, 3, 5, 7, and 10 dpa), at least 20 independent trunk fragments were collected for morphological observation. For qPCR and RNA-seq assays, three biological replicates (≥5 planarians pooled per replicate) were prepared.

Newly regenerated area quantification: Bright-field images of regenerating trunk fragments were captured at unified magnification. The outer boundary of the newly regenerated region was manually outlined using ImageJ software; pixel area was converted to actual tissue area, and blastema regeneration rate was calculated as (newly regenerated area/total trunk fragment area). Five biological replicates were quantified for each time point per group.

### WISH (whole mount *in situ* hybridization)

WISH experiments were performed as previously described (Zhen et al., 2020). All probe amplification primer sequences used in WISH are listed in Supplementary Table S1. In brief, hybridization reactions were performed by incubating samples with the antisense RNA probe (1 ng/μL) at 56 °C for 16 h. Then, the samples were rinsed with maleic acid buffer (MABT, Sigma-Aldrich), followed by a 2 h blocking step with 10% horse serum at room temperature. Next, antibodies (anti-DIG-AP, 1:2000, Roche) diluted in Tween 20-supplemented MABT containing 10% horse serum were added for subsequent WISH. The samples were subsequently examined under a microscope (Stereomicroscope, Nikon P-DSL32, China; Leica Microsystems CMS GmbH, Germany) and documented. *TH*-positive (red dashed box), *TPH*-positive and *GAD*-positive dots were quantified with the particle analysis module of ImageJ 6.0. For *Chat* signal quantification (red dashed box), positive signals were quantified within the cephalic-ganglia region demarcated by red dashed lines.

### RNA interference

The dsRNAs corresponding to *DSP1* and *CnB* were generated via the MEGAscript™ RNAi Kit purchased from Invitrogen (USA). Planarians assigned to the experimental group were immersed in a dsRNA solution at a concentration of 40 ng/μL for 6 h prior to their transfer into the designated culture medium. In parallel, planarians in the control group were treated with GFP-targeting dsRNA under the same conditions.

### Immunohistochemistry

Immunostaining was conducted following our established protocol (Zhen et al., 2020). Briefly, planarians were euthanized in 2% HCl, fixed in 4% paraformaldehyde (PFA, Sigma) diluted in PBS at 37 °C for 1 h, and dehydrated in anhydrous methanol. Samples were incubated with primary antibodies: Anti-SYNAPSIN (clone 3C11, 1:300, DSHB, USA) and Anti-phosphohistone-H3 (S10) (hereafter H3P) (1:200, Cell Signaling Technology, USA). Samples were then incubated overnight at 4 °C with fluorophore-conjugated secondary antibodies: Alexa Fluor 594-conjugated rabbit anti-mouse IgG (1:200, EarthOx, USA) and goat anti-rabbit IgG (1:200, EarthOx, USA). Immunofluorescent signals were visualized and captured using a laser scanning confocal microscope (Zeiss LSM900).

### Quantitative real-time PCR (qPCR)

Briefly, total RNA was isolated using TRIzol reagent (Invitrogen, USA). qPCR amplifications were performed on a qTower 3G real-time PCR system (Analytikjena, Germany) with ChamQ Universal SYBR qPCR Master Mix (Q711–03, Vazyme, China) at an annealing temperature of 55 °C. Relative expression levels, normalized to *Gapdh*, were calculated via the 2^−ΔΔCt^ method.

### RNA-seq analysis

Three days after the final round of dsRNA treatment (3 days post RNAi), trunk fragments from *DSP1* RNAi and control groups were harvested for total RNA extraction and subsequent transcriptome sequencing. Raw paired-end reads were trimmed and subjected to quality control via fastp using default settings. The resultant clean reads of each sample were subsequently adopted for *de novo* transcriptome assembly with Trinity. To further improve assembly integrity, all assembled contigs were filtered sequentially using CD-HIT and TransRate, followed by quality evaluation via BUSCO (Benchmarking Universal Single-Copy Orthologs). GO and KEGG enrichment analyses were applied for functional exploration of DEGs, and enriched items with Bonferroni-adjusted P < 0.05 were screened against the whole-transcriptome background. Goatools and Python SciPy were separately applied for GO and KEGG analysis, and all data analysis was finished on Majorbio Cloud platform. The raw RNA-seq data supporting the findings of this study are available in the NCBI Sequence Read Archive (SRA) repository with accession numbers PRJNA1483247 and SAMN61176826. Heatmaps were generated using the R programming language combined with fastcluster software. For the target gene set, clustering was based on the log_2_-transformed expression ratios between pairwise samples; inter-gene distances were computed via a corresponding algorithm and iteratively optimized to classify genes into distinct subclusters.

### Statistical analysis

Statistical analyses were carried out using SPSS statistical software for Windows, version 16.0 (SPSS). For comparisons among ≥3 experimental groups (Control, *DSP1* RNAi, *CnB* RNAi, *DSP1*+*CnB* double RNAi), one-way ANOVA followed by Tukey’s multiple comparison *post hoc* test was adopted, rather than independent sample t-test. Two-group comparisons only adopt Student’s t-test. All datasets were verified to conform to normal distribution and homogeneity of variance before statistical analysis. Data are presented as mean ± SEM; *p < 0.05, **p < 0.01 were defined as statistically significant.

## Results

### Expression of *DSP1* in the intact and regenerative planarians

Using an optimized WISH protocol (see Materials and Methods), we detected robust *DSP1* expression in intact planarians. Strong signals were predominantly localized to the parenchyma, retropharyngeal region (referring to the mesenchymal parenchyma posterior to the pharyngeal pouch), and cephalic ganglia, as indicated by the arrows; epidermal cells show almost no specific staining, pharynx epithelial cells have weak background signals only (Figure 1A). qPCR analysis of trunk and tail regenerates at 1, 3, 5, 7, and 10 days post amputation (dpa) revealed dynamic temporal changes in *DSP1* expression during regeneration. Specifically, *DSP1* transcript levels decreased from 1 to 3 dpa, elevated at 5 dpa, and decreased again at 7 dpa and finally increased again by 10 dpa (Figure 1B). WISH analysis further indicated that no positive signal was detected at the regeneration site at 1 dpa. By 3 dpa, *DSP1* expression became restricted exclusively to the emerging brain primordia rather than the whole blastema in both trunk and tail regenerates (Figure 1C, arrows). As regeneration progressed, *DSP1* signals progressively accumulated in newly regenerated neural tissues. These spatiotemporal expression patterns collectively suggest that *DSP1* may be critically involved in planarian neural regeneration, rather than general blastema-wide regeneration.

**FIGURE 1:**
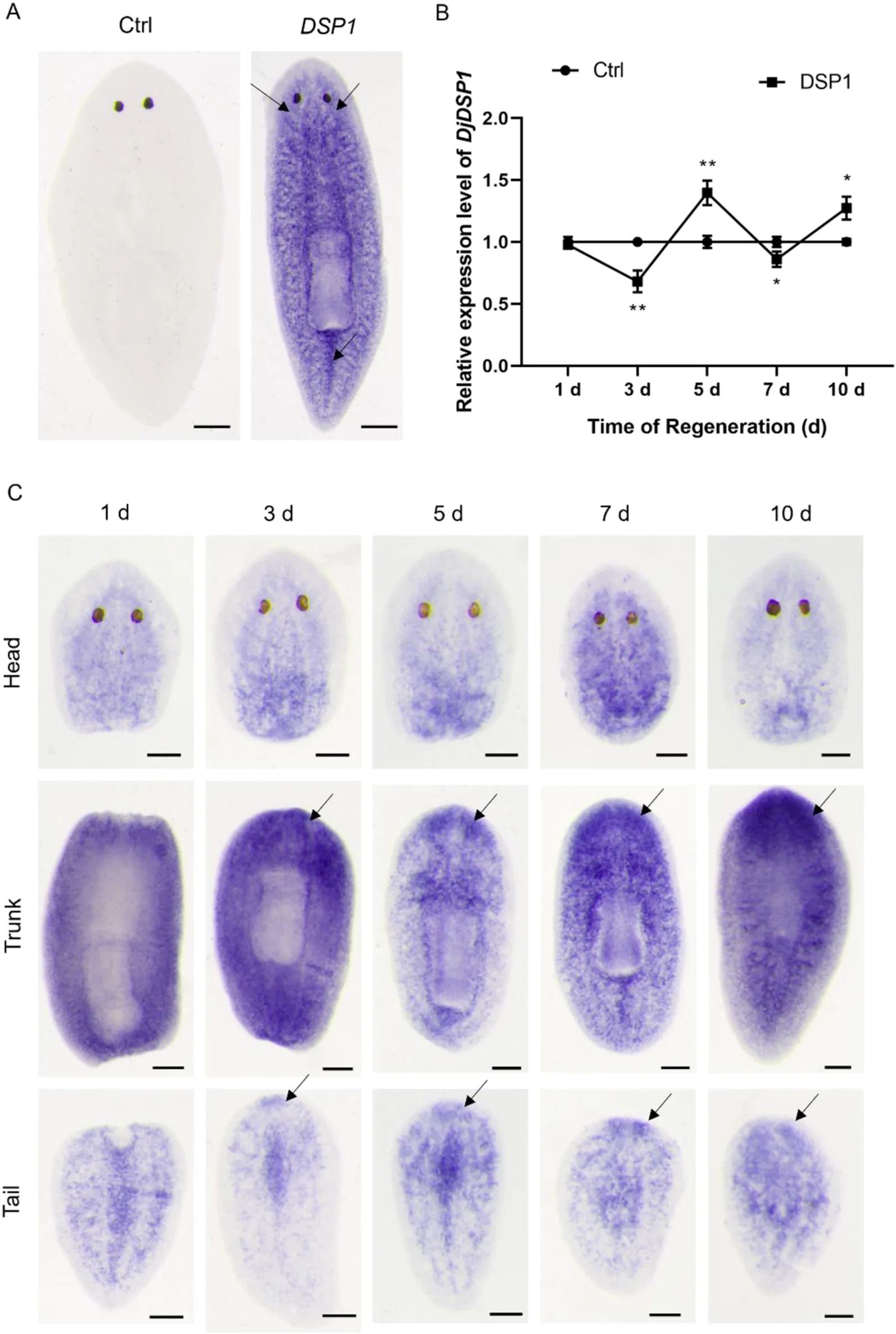
Spatiotemporal expression of *DSP1* in planarians **(A)** Expression analysis of *DSP1* in the intact planarians by WISH. Scale bars: 250 μm. Arrows indicate *DSP1*-positive signals in cephalic ganglia. **(B)** qPCR analysis of *DSP1* expression the regenerated planarians at 1, 3, 5, 7, 10 dpa. N = 5. *p < 0.05, **p < 0.01. **(C)** Expression analysis of *DSP1* at 1, 3, 5, 7 and 10 dpa. N = 5. Scale bars: 250 μm. Arrows indicate *DSP1*-positive signals in regenerating brain nerve.

### Knockdown of *DSP1* causes accelerated planarian regeneration

To clarify the physiological functions of *DSP1*, we conducted RNAi using a soaking method that is widely used in planarian research (Orii et al., 2003). As demonstrated in Figures 2A,B, three rounds of *DSP1* RNAi treatment significantly reduced the expression of *DSP1* in *D. japonica*.

**FIGURE 2:**
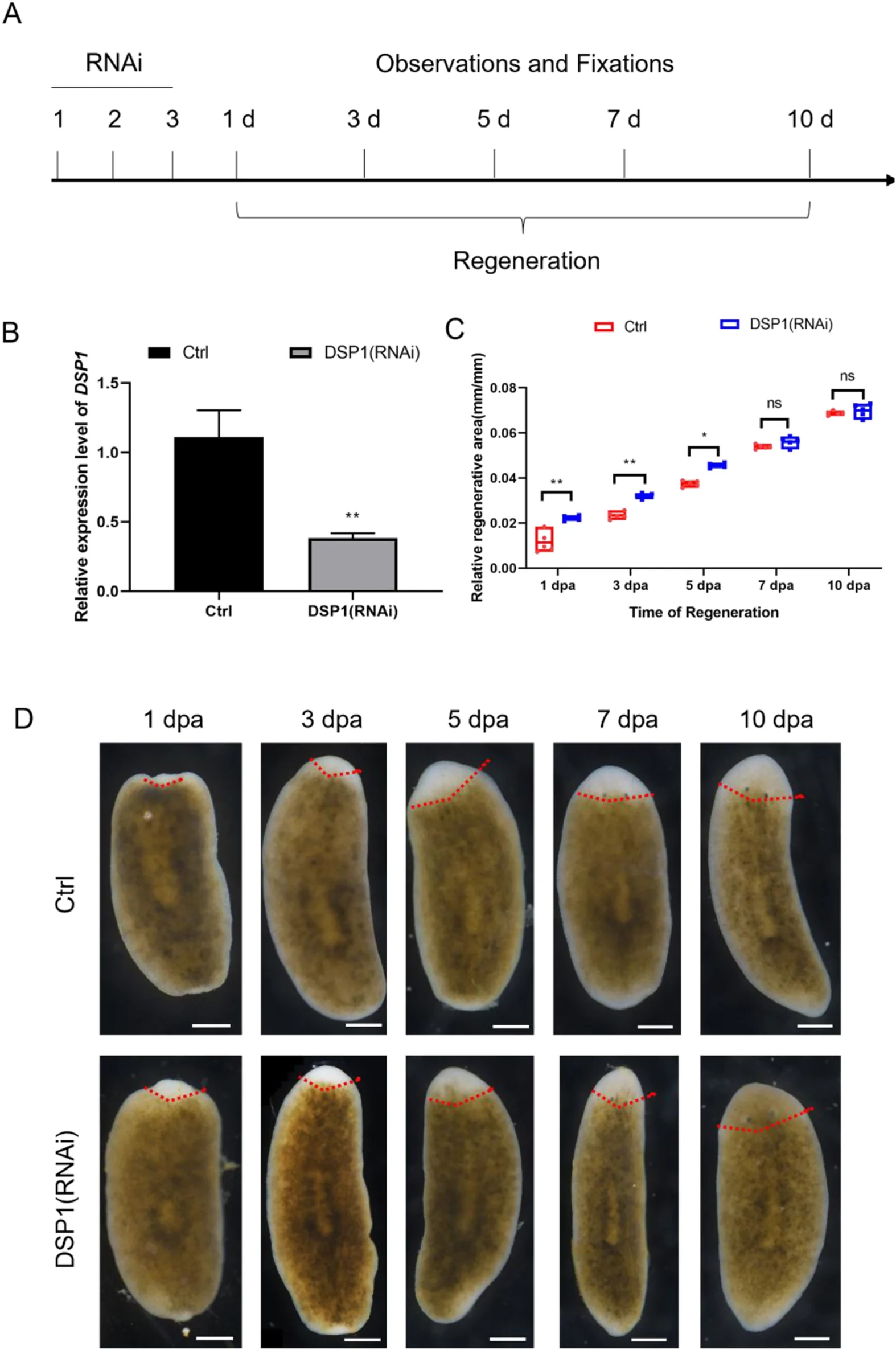
Knockdown of *DSP1* significantly accelerates the regeneration process in planarians. **(A)** Flowchart of RNAi and amputation schedules for this study. **(B)** qPCR analysis of RNAi efficiency after *DSP1* RNAi. N = 5. **p < 0.01. **(C)** Quantification of the area of newly regenerated head tissue at different time-points, n = 5. *p < 0.05, **p < 0.01. ns, not significant. **(D)** Representative images of morphologic changes in the regenerated planarians at 1, 3, 5, 7 and 10 dpa after *DSP1* RNAi. Scale bars: 250 μm. N = 20. Red dotted lines are clarified to outline the newly regenerated region.

To explore the function of *DSP1* during regeneration, we amputated the head and tail of planarians on days 2 following the final RNAi treatment, and collected regenerating trunk fragments for observation at 1, 3, 5, 7 and 10 dpa. As shown in Figures 2C,D, quantitative measurement using ImageJ revealed that *DSP1*-knockdown planarians developed remarkably larger newly regenerated area than control individuals at one dpa. At three dpa, the brain primordia were also enlarged in the *DSP1* RNAi group compared with controls, as determined by measuring the newly regenerated area. Over time, regeneration in the RNAi group gradually became complete. At 10 dpa, head pigmentation returned to the normal physiological state, while newly generated head tissue in the control group remained unpigmented. Restoration of head pigmentation depends on neoblast differentiation into pigment-producing cells (He et al., 2017). Earlier pigment recovery in *DSP1*-knockdown planarians indicated accelerated neoblast differentiation toward pigment cell subtypes alongside enhanced neural regeneration. Collectively, these results indicate that *DSP1* knockdown accelerates planarian head regeneration.

To further investigate whether *DSP1* affects planarian regeneration, we examined the nervous system regeneration via WISH (*Chat* (Choline acetyltransferase, marker of cholinergic neurons), *TH* (Tyrosine hydroxylase, marker of dopaminergic neurons)) and immunofluorescence (Anti-SYNAPSIN, the pan-neuronal marker anti-SYNORF1 (3C11) targeting synapsin was used to label the whole planarian CNS). As shown in Figure 3A, neural regeneration proceeded more thoroughly in *DSP1*-knockdown planarians relative to controls. Specifically, regenerating cephalic ganglia exhibited extended neurite outgrowth in *DSP1* knockdown animals. At 7 dpa, cephalic ganglia expanded further, and lateral nerve outgrowth was clearly observed in knockdown individuals. At 10 dpa, cephalic ganglia were still undergoing regeneration in both groups; nevertheless, the ganglia and lateral nerve fibers of the *DSP1* RNAi group were more developed and prominent. Consistent morphological improvement of *Chat* ^+^ and *TH* ^+^ neurons was validated by *Chat* and *TH* WISH assays (Figures 3B,C). Quantitative analysis of discrete *Chat* ^+^ and *TH* ^+^ signal dots confirmed that the number of *Chat* ^+^- and *TH* ^+^-positive dots was significantly elevated in the *DSP1* RNAi group at 5, 7 and 10 dpa, consistent with the morphological observations from WISH (Figures 3D,E). Collectively, these data confirm that *DSP1* functions as an inhibitory regulator during planarian central nervous system regeneration.

**FIGURE 3:**
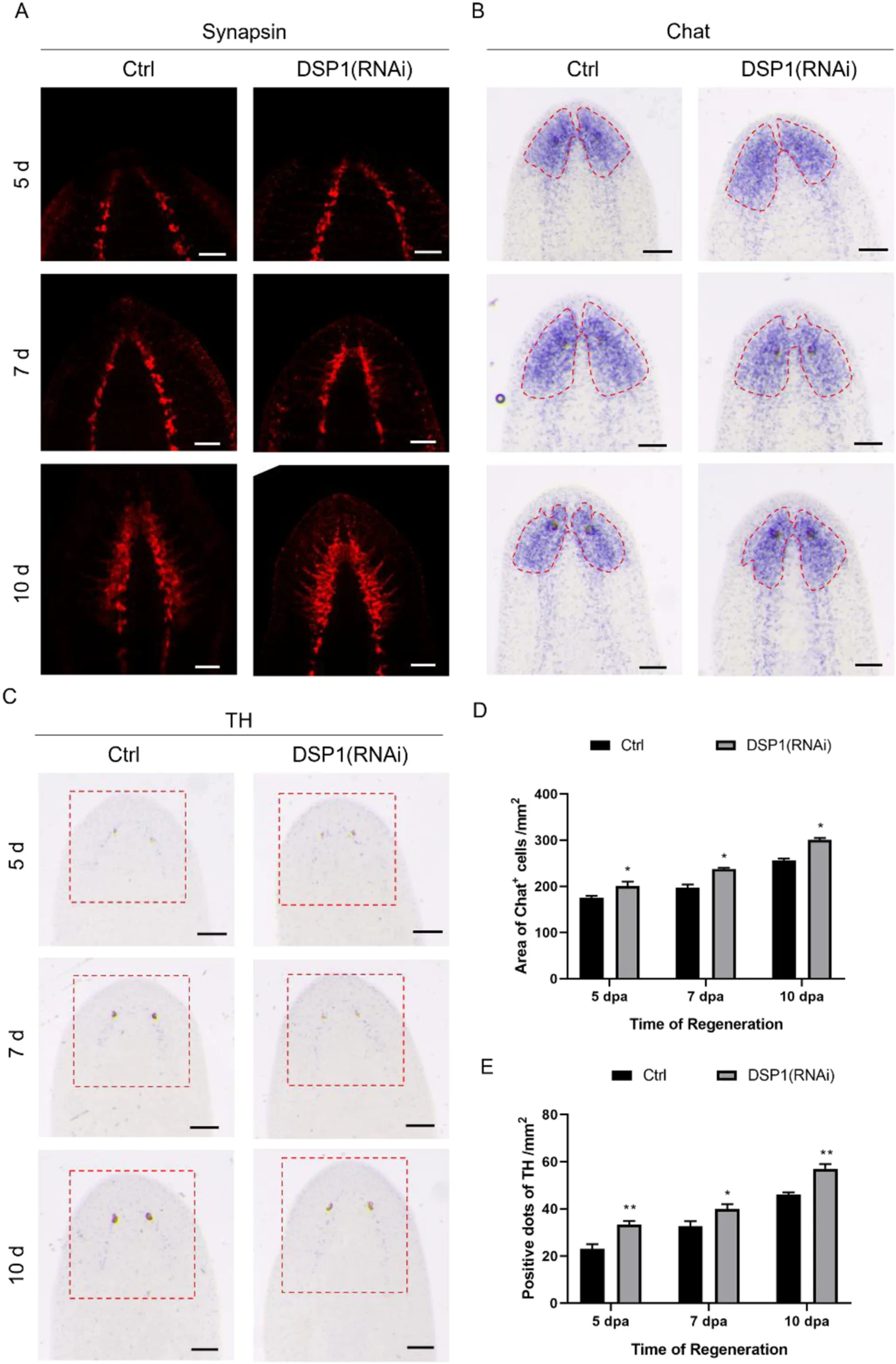
Knockdown of *DSP1* significantly accelerates the brain nerve regeneration in planarians. **(A)** Whole-mount immunohistochemistry using anti-SYNAPSIN antibodies was performed on regenerating planarians at 5, 7, and 10 dpa following *DSP1* RNAi. Scale bars: 100 μm. **(B)** WISH against *Chat* was performed on regenerating planarians at 5, 7, and 10 dpa following *DSP1* RNAi. Scale bar: 250 μm. **(C)** WISH against *TH* was performed on regenerating planarians at 5, 7, and 10 dpa following *DSP1* RNAi. Scale bar: 250 μm. **(D)** Area of *Chat* ^+^ neurons (n = 5). **(E)** Positive dots of *TH* ^+^ neurons (n = 5). **p < 0.01, *p < 0.05. Red dashed-line regions represent the measured areas.

### Knockdown of *DSP1* affects neural stem cells

Abnormal neural regeneration phenotypes are generally attributed to defects in pluripotent stem cells or neural stem cells. To explore *DSP1*’s role in planarian head regeneration, we assessed the expression of the neoblast marker *DjPiwiA* in regenerating trunk fragments via WISH and qPCR. As shown in Supplementary Figures S1A,B, knockdown of *DSP1* exerted no obvious influence on *DjPiwiA* transcript abundance at 1, 3, 5, 7 and 10 dpa relative to control groups, revealing that *DSP1* depletion does not alter the overall abundance or self-renewal capacity of neoblasts, the primary proliferative pluripotent cell population in planarians. Subsequently, we performed immunofluorescence staining for the mitotic marker H3P to clarify whether *DSP1* modulates stem cell proliferation, with detection conducted at 1, 2, 3 and 5 dpa post RNAi treatment. Consistent with published studies, the mitotic peak occurring as part of a normal wound response at 2 dpa was significantly increased after *DSP1* silencing. Following the proliferation peak, mitotic activity decreased in both groups over time. Nevertheless, the *DSP1* RNAi group maintained higher mitotic activity at all detected time-points relative to controls (Supplementary Figures S1C,D). These findings reveal that *DSP1* knockdown enhances neoblast mitotic activity without expanding the overall neoblast pool. More progeny cells are driven toward differentiation, thereby giving rise to altered expression of differentiation-associated markers.

### Downregulation of *DSP1* led to an increased number of early neoblasts progeny cells during planarian head regeneration

To determine the role of *DSP1* in differentiation in planarians, we screened differentiation-related marker genes based on RNA-seq data collected from 1 dpa regenerative trunk fragments. The selected markers covered early neoblast progeny (*NB.21*), late neoblast progeny (*Agat-1*), neural stem cells (*Ston2*), and mature neuron subtypes (*Chat*, *GAD, TH*, *TPH*, *Neuropeptide F*). RNA-seq results revealed heterogeneous expression changes of these differentiation markers upon *DSP1* knockdown: some markers were upregulated, whereas others were downregulated (Figure 4E). Notably, for several genes including *Agat-1*, *GAD* and *TPH*, we observed inconsistent trends between bulk RNA-seq and follow-up qPCR and WISH assays. Such discrepancy may arise from cell-composition effects: altered proportions of neural and progenitor cell populations within regenerating fragments can shape bulk transcriptome signals, even when target gene expression is elevated within the corresponding cell-types. Subsequent qPCR verification further validated that the expression trends of *NB.21* and *Ston2* matched the transcriptome profiling outcomes (Figure 4D). As illustrated in Figures 4A,B, the expression of *NB.21* and *Agat-1* was significantly increased in *DSP1* RNAi-treated planarians. We further applied WISH targeting the neural stem cell marker *Ston2* to explore *DSP1*’s influence on neuronal differentiation. At 3 dpa, brain primordia were markedly enlarged in the *DSP1* knockdown group relative to controls (Figure 4C), suggesting altered neuronal differentiation after *DSP1* silencing.

**FIGURE 4:**
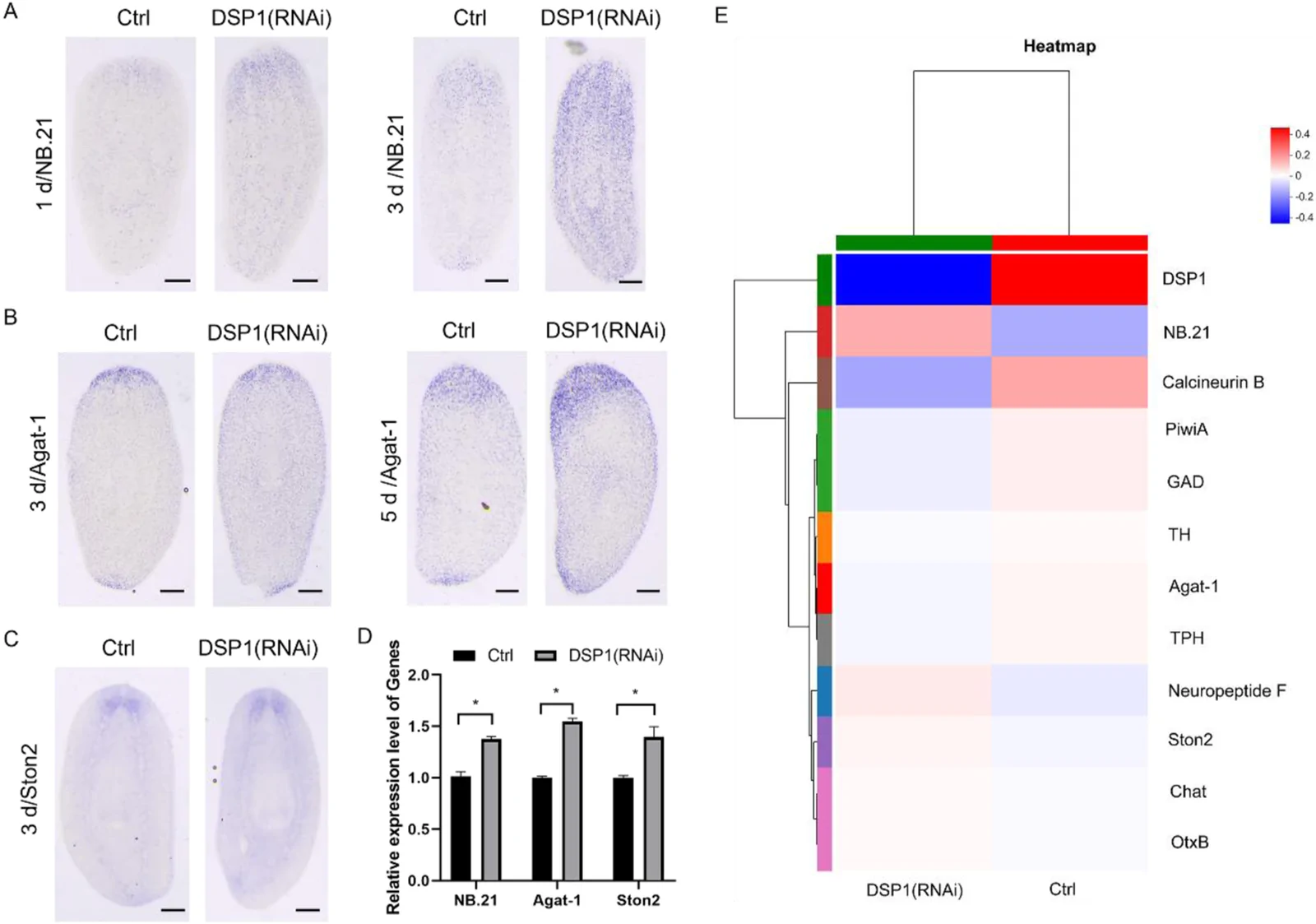
Knockdown of *DSP1* affects differentiation marker genes expression **(A)** Expression of *NB.21* examined by WISH in the regenerated planarians at one dpa and three dpa after *DSP1* RNAi. Scale bar: 250 μm **(B)** Expression of *Agat-1* examined by WISH in the regenerated planarians at three dpa and five dpa after *DSP1*RNAi. Scale bar: 250 μm. **(C)** Expression of *Ston2* examined by WISH in the regenerated planarians at three dpa after *DSP1* RNAi. Scale bar: 250 μm. **(D)** qPCR of the *NB.21*, *Agat-1* and *Ston2* in the regenerated planarians at three dpa after *DSP1* RNAi. n = 5. *p < 0.05. **(E)** Heatmap of genes expression of *DSP1*, *NB.21*, *CnB*, *GAD*, *TH*, *Agat-1*, *TPH*, *Neuropeptide F*, *Ston2*, *Chat* and *OtxB* in the regenerated planarians at one dpa after *DSP1* RNAi, in comparison with that treated with control RNAi.

### Transcriptome profiling upon *DSP1* knockdown identifies *CnB* as a downstream candidate gene

To further elucidate how *DSP1* modulates head regeneration in planarians, RNA-seq was conducted on planarians after *DSP1* RNAi knockdown after the final round of dsRNA treatment. A total of 915 differentially expressed genes (DEGs) were identified via RNA-seq profiling, with 696 transcripts upregulated and 219 downregulated (Figures 5A,B). Among these transcriptional changes, *CnB* was found to be downregulated. *CnB* is the regulatory subunit of calcineurin, a core effector of non-canonical Wnt/Ca^2+^ signaling, which our lab previously confirmed regulates planarian GABAergic neuron regeneration, suggesting its potential requirement for planarian head regeneration (Zhen et al., 2020; Zhen et al., 2022).

**FIGURE 5:**
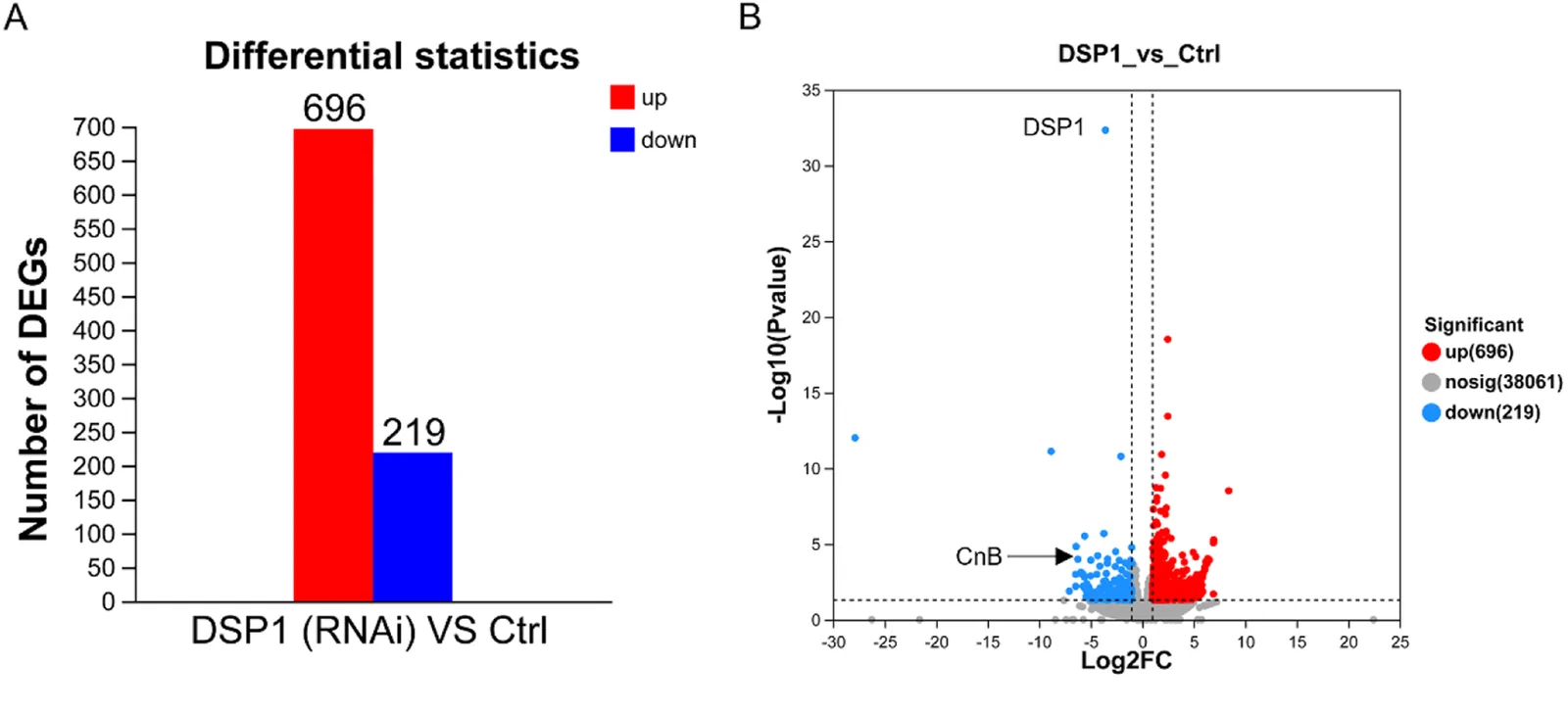
RNA-seq analysis after knockdown of *DSP1* **(A, B)** The number of DEGs identified after *DSP1* RNAi, with a threshold of the ratio change ≥2 and a q-value of <0.05. The red and blue columns represent up- and downregulated genes in planarians after *DSP1* RNAi.

### Co-knockdown of *DSP1* and *CnB* led to a modest acceleration of planarian regeneration relative to single *DSP1* knockdown

To explore whether the regenerative phenotypes triggered by *DSP1* knockdown rely on *CnB* mRNA changes, we established three experimental groups via RNAi: *DSP1* single knockdown, *CnB* single knockdown, and *DSP1* and *CnB* co-knockdown. Notably, *CnB* knockdown alone produced no obvious influence on central nervous system regeneration (Figure 6; Supplementary Figure S2). Compared with *DSP1* single knockdown, *DSP1* and *CnB* co-knockdown moderately accelerated overall regeneration progress. At 3 dpa, co-knockdown groups displayed enlarged brain primordia (Figures 7B,C). At 5, 7 and 10 dpa, the nerve fiber network derived from the double-knockdown group exhibits more extensive lateral extension and denser neural meshwork compared with the single *DSP1* knockdown group at the same regeneration time point (Figure 7A). In addition, co-knockdown resulted in slightly higher counts of *TH* ^+^, *TPH* ^+^ and *GAD* ^+^ neurons than *DSP1* single knockdown (Figure 8). Collectively, these results demonstrate that *DSP1* cooperates with *CnB* to jointly modulate cephalic ganglia regeneration in planarians (Figure 9).

**FIGURE 6:**
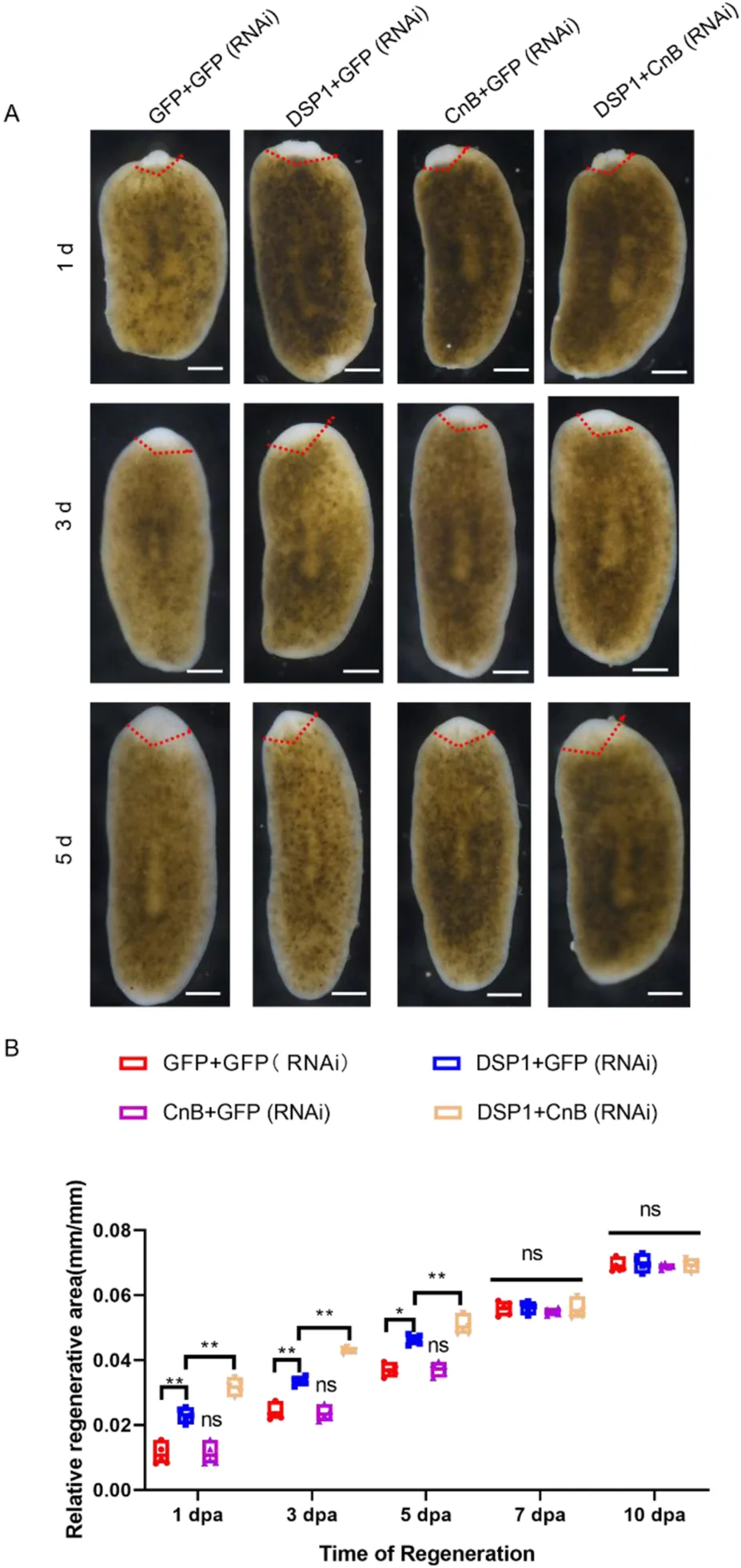
Co-knockdown of *DSP1* and *CnB* significantly accelerates the regeneration process in planarians. **(A)** Representative images of morphologic changes in the regenerated planarians at 1, 3, and five dpa after Control RNAi, *DSP1* RNAi, *CnB* RNAi and *DSP1*+*CnB* RNAi. Scale bars: 250 μm. n = 20. **(B)** Quantification of newly regenerated area, n = 5. *p < 0.05, **p < 0.01. Ns, not significant.

**FIGURE 7:**
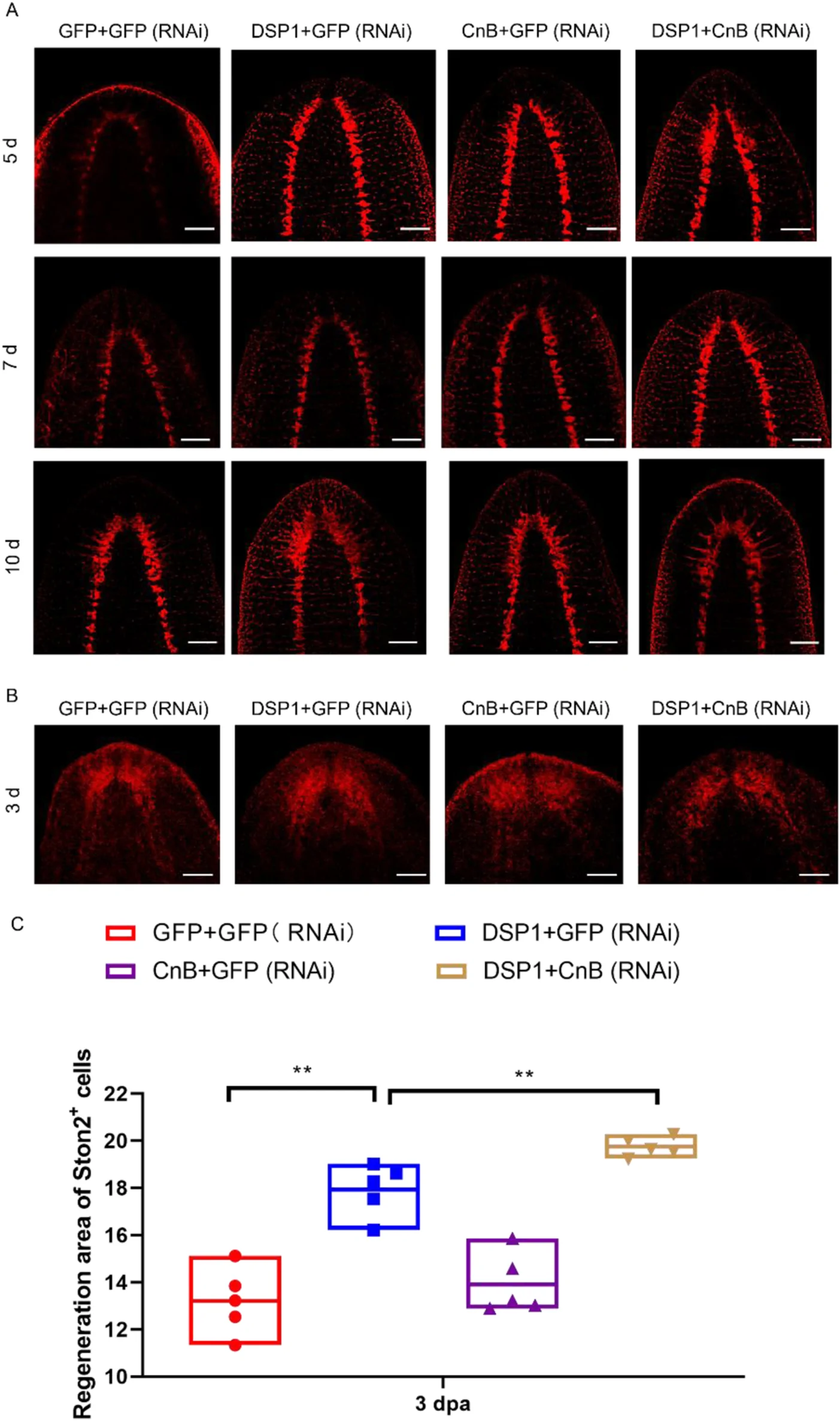
Co-knockdown of *DSP1* and *CnB* significantly accelerates the brain nerve regeneration in planarians. **(A)** Whole-mount immunohistochemistry with antibodies against SYNAPSIN in the regenerated planarians at 5, 7 and 10 dpa after either *DSP1* and *CnB* RNAi alone or *DSP1*+ *CnB* double RNAi. Scale bars: 100 μm. **(B)** FISH of *Ston2* in the regenerated planarians at three dpa after either *DSP1* and *CnB* RNAi alone or *DSP1*+ CnB double RNAi. Scale bar: 100 μm. **(C)** Quantification of blastema regeneration area of *Ston2* ^*+*^ cells, n = 5. **p < 0.01.

**FIGURE 8:**
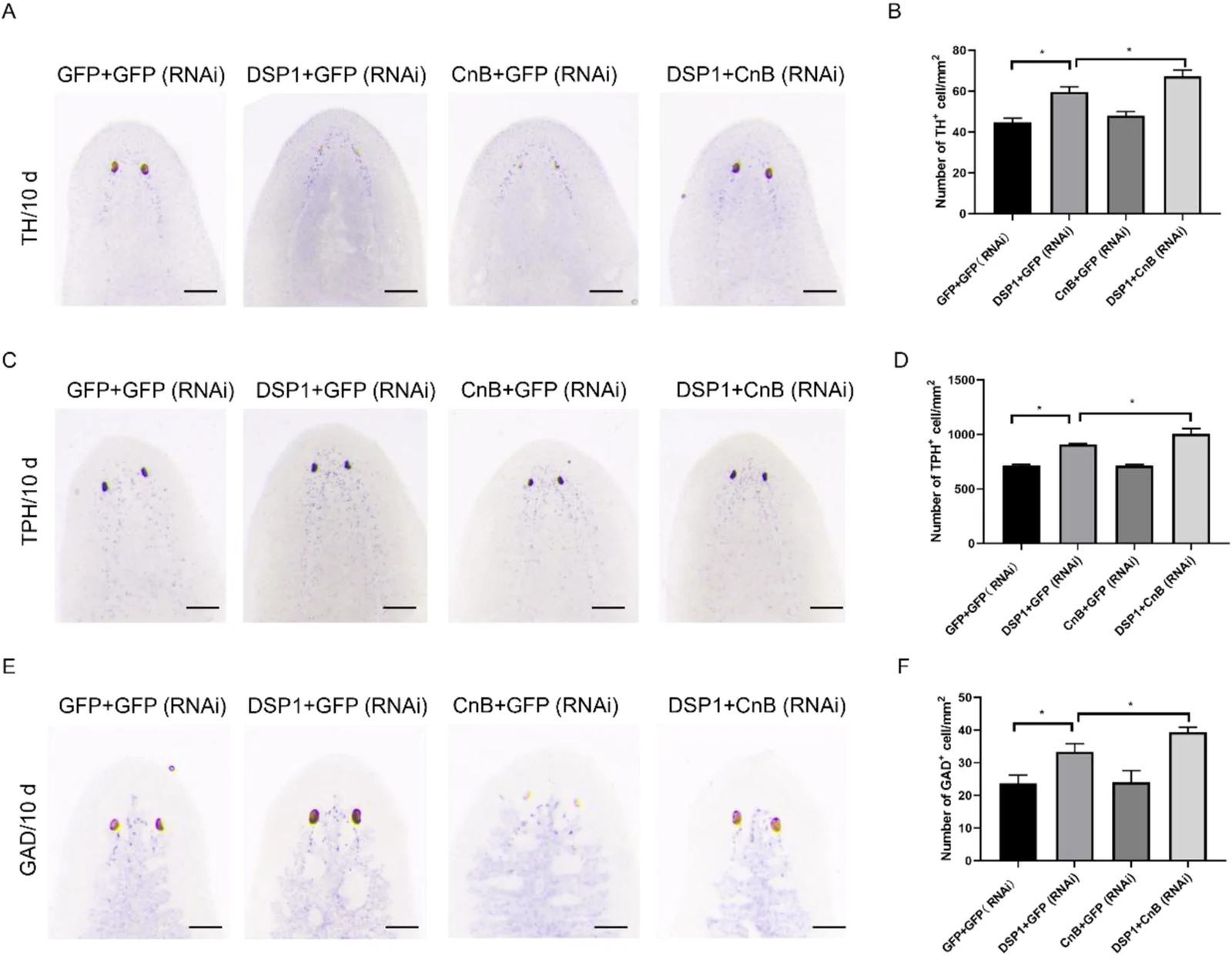
WISH of different neurons **(A)** WISH of *TH* in the regenerated planarians at seven dpa after either *DSP1* and *CnB* RNAi alone or *DSP1*+ CnB double RNAi. Scale bar: 250 μm. **(B)** Quantification of regeneration area of *TH* ^*+*^ cells, n = 5. *p < 0.05. **(C)** WISH of *TPH* in the regenerated planarians at seven dpa after either *DSP1* and *CnB* RNAi alone or *DSP1*+ CnB double RNAi. Scale bar: 250 μm. **(D)** Quantification of regeneration area of *TPH* ^*+*^ cells, n = 5. *p < 0.05 **(E)** WISH of *GAD* in the regenerated planarians at seven dpa after either *DSP1* and *CnB* RNAi alone or *DSP1*+ CnB double RNAi. Scale bar: 250 μm. **(F)** Quantification of regeneration area of *GAD* ^*+*^ cells, n = 5. *p < 0.05.

**FIGURE 9:**
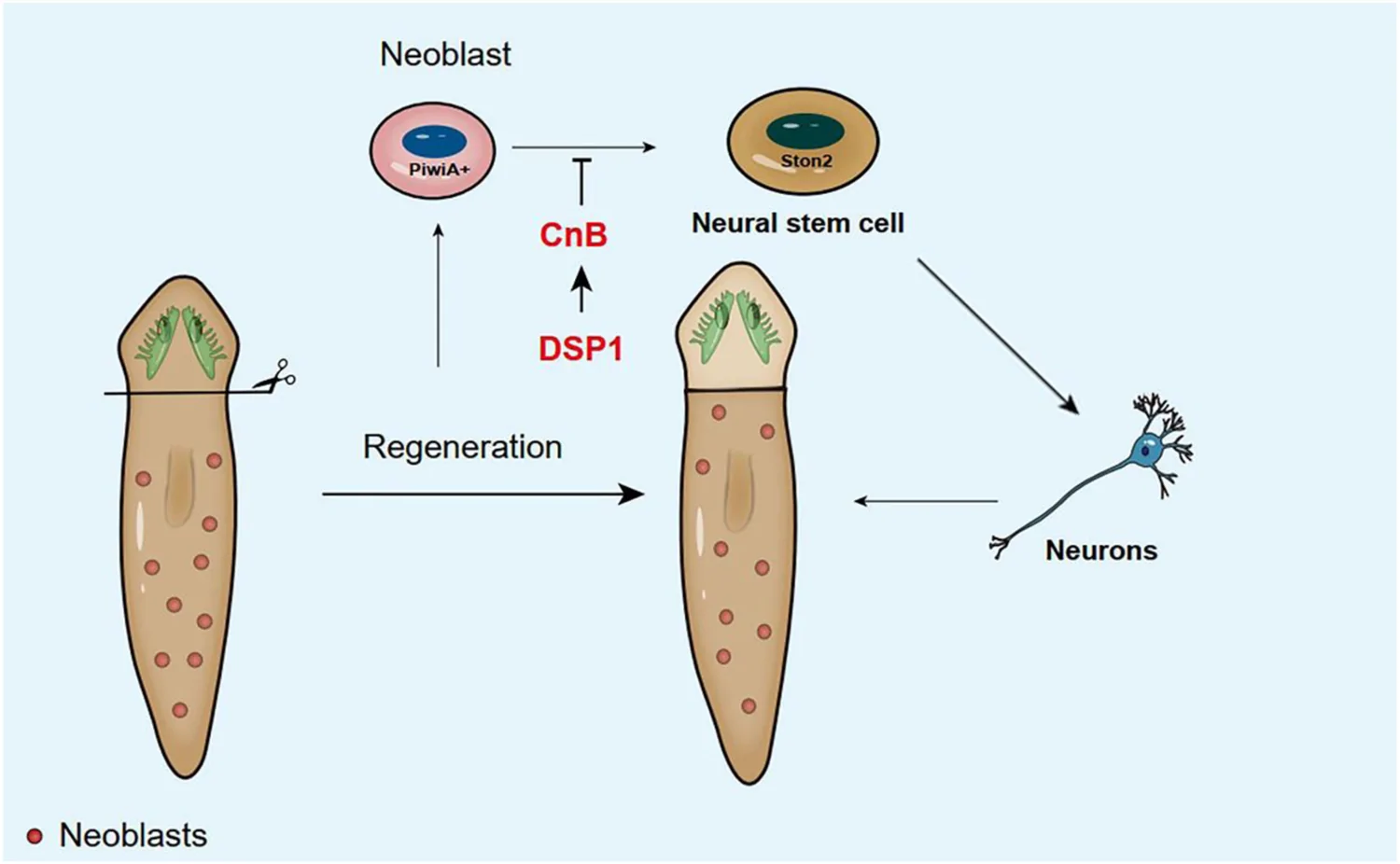
Proposed models illustrating the essential role of DSP1/CnB signaling in planarian cranial nerve regeneration *DSP1* as a conserved key regulator that modulates neural stem cell function partly depends on *CnB* to govern neuronal regeneration.

## Discussion

Planarians serve as an ideal *in vivo* model for exploring stem cell biology and neural regeneration. In this study, we found that *DSP1* was widely expressed in the intact planarian, predominantly in parenchyma, retropharyngeal and cerebral nerves. Knockdown of *DSP1* accelerates blastema formation at 1 dpa without altering the overall neoblast pool. *DSP1* silencing accelerates brain regeneration and elevates the abundance of differentiated stem cell progeny, alongside the transcriptional downregulation of *CnB*, a key component of the Wnt signaling pathway. Notably, co-knockdown of *DSP1* and *CnB* generates an enhanced regenerative phenotype and further facilitates blastema growth. Collectively, our findings demonstrate that there is a genetic interaction between *DSP1* and *CnB* to regulate cephalic ganglia regeneration in planarians.

DSP1, the invertebrate ortholog of vertebrate high-mobility group box 1(HMGB1), is highly conserved among eukaryotes. It was first identified in 1994 as a transcriptional co-repressor of Dorsal in *D. melanogaster* (Lehming et al., 1994; Bianchi, 2009). Encoded by the *DSP1* gene, this protein consists of a glutamine-rich N-terminal domain, an acidic C-terminal tail, and two conserved HMG-box motifs (HMG box A and HMG box B) (Canaple et al., 1997). Previous studies have validated *DSP1* expression in the ovaries and brain of adult *Drosophila*, highlighting its essential functions during embryonic development (Lehming et al., 1994). Consistent with these conserved characteristics, our WISH results confirm ubiquitous *DSP1* expression in intact planarians, with prominent enrichment in the parenchyma, retropharyngeal tissues and cerebral nerve ganglia. Furthermore, *DSP1* transcript levels undergo obvious dynamic fluctuations throughout planarian regeneration (Figure 1). Collectively, these lines of evidence support the notion that *DSP1* may act as a crucial regulator of planarian cephalic ganglia regeneration.

DSP1 plays a critical role in DNA binding including transcription factors and chromatin remodeling complexes (Stros et al., 2007). Previous studies have verified that DSP1 binds Dorsal and regulates its transcriptional activity; Dorsal serves as a master transcription factor governing dorsoventral patterning during *Drosophila* embryonic development (Lehming et al., 1994; Brickman et al., 1999). Additionally, HMGB1 (the mammalian ortholog of DSP1) is closely associated with multiple neurodegenerative disorders, including Parkinson’s disease (PD), multiple sclerosis (MS), and amyotrophic lateral sclerosis (ALS) (Ikram et al., 2022). DSP1 overexpression impairs climbing ability and shortens lifespan, accompanied by neuromuscular junction (NMJ) defects, microphthalmia, and fewer TH-positive neurons. These phenotypes collectively confirm the neuronal toxicity triggered by excess *DSP1* (Baek et al., 2024). Combining phenotypic observation and molecular marker detection, we identify a two-tier regulatory pattern through which *DSP1* modulates planarian pluripotent stem cells and their neural progeny. The pan-neoblast marker *PiwiA* displays equivalent expression levels between control and *DSP1*-knockdown groups throughout 1–10 dpa, revealing that *DSP1* depletion does not alter the overall abundance or self-renewal capacity of neoblasts, the primary proliferative pluripotent cell population in planarians. Notably, elevated H3P mitotic signals in *DSP1*-knockdown animals reflect increased mitotic activity of neoblasts, rather than an expansion of the total neoblast pool (Zeng et al., 2018). On the contrary, both WISH and qPCR verify remarkable upregulation of three lineage differentiation markers after *DSP1* silencing, including the early neoblast progeny marker *NB.21*, late differentiated progeny marker *Agat-1*, and neural stem cell marker *Ston2*. Under normal physiological conditions in intact planarians, endogenous *DSP1* serves as a transcriptional repressor to constrain the directed differentiation of neoblasts into neural progenitors. Once this inhibitory effect is eliminated via *DSP1* knockdown, the transformation from neoblasts to neural precursors speeds up, eventually expanding brain primordia and increasing the production of diverse neuronal subtypes within regenerative blastemas. We also note that bulk RNA-seq data should be interpreted with caution in regenerating planarians, since cell-composition effects may mask cell-type-specific transcriptional changes.

Although our study described morphological regeneration of planarian cephalic ganglia and multiple neuronal subtypes, we did not perform behavioral tests to verify neural circuit functional recovery. Locomotor and phototaxis assays are classic quantitative indicators to judge the intactness of regenerated nervous systems, including photoreception, motor coordination and neuron maturation. Future work will adopt these behavioral detection methods to compare sensory and motor functional recovery among control, *DSP1* RNAi, *CnB* RNAi and double knockdown planarians.

In planarians, the canonical Wnt signal pathway regulated anteroposterior axis regeneration via β-catenin gradients, and non-canonical Wnt mediated mediolateral patterning and neural regeneration (Gurley et al., 2008; Petersen, 2023). Calcineurin (CaN) is a eukaryotic Ca^2+^/calmodulin-dependent serine/threonine phosphatase that forms a heterodimer composed of a catalytic subunit A and a myristoylated regulatory subunit B (Rusnak and Mertz, 2000). *CaN* is widely distributed in neuronal subcellular compartments (Jiang et al., 2012). As the regulatory subunit of calcineurin, *CnB* modulates the *NFAT* signaling cascade to activate the transcription of genes responsible for neuronal migration; this regulatory process is essential for normal cerebral cortex and cerebellum development (Kipanyula et al., 2016). Our transcriptome sequencing and qPCR results confirmed that *DSP1* positively modulates the transcription of *CnB*; *DSP1* RNAi induces significant downregulation of endogenous *CnB*, which relieves the intrinsic inhibitory constraint on neural lineage differentiation and accelerates cephalic ganglia reconstruction. Moreover, simultaneous knockdown of *DSP1* and *CnB* produced synergistic enhancement of blastema expansion, brain primordia growth, and the production of *TH* ^+^ dopaminergic, *TPH* ^+^ serotonergic and *GAD* ^+^ GABAergic neurons. This co-knockdown phenotype solidifies that *CnB* functions as an indispensable downstream effector required for *DSP1*-mediated suppression of neural regeneration.

In conclusion, our study identified planarian *DSP1* as a conserved key regulator that modulates neural stem cell function partly depends on *CnB* to govern neuronal regeneration (Figure 9). These findings a theoretical basis for understanding the DSP1/CnB signaling axis in regulating neural regeneration in mammals, and have important implications for the development of drugs targeting neuronal regeneration. Due to technical limitation of planarian overexpression system at present, we will construct a *DSP1* overexpression system for *D. japonica* in the future research project to directly validate the bidirectional regulatory function of *DSP1*.

## Data Availability

The datasets presented in this study can be found in online repositories. The names of the repository/repositories and accession number(s) can be found below: https://www.ncbi.nlm.nih.gov/bioproject/PRJNA1483247 https://www.researchsquare.com/article/rs-9686603/v1, DOI: https://doi.org/10.21203/rs.3.rs-9686603/v1.
