# Supplementary material for: DSP1 regulates planarian (Dugesia japonica) neural regeneration depending on CnB
Source: Front Cell Dev Biol. 2026 Oct 8;14:1932725. doi: 10.3389/fcell.2026.1932725 (PMC13688025; doi:10.3389/fcell.2026.1932725)
Supplement: Supplementary file 1 [file DataSheet1.docx]

Figure S1 Knockdown of *DSP1* affect neoblasts

(A)	WISH of *PiwiA* in the regenerated planarians at 1, 3, 5, 7 and 10 dpa after *DSP1* or control RNAi. Scale bar: 250 μm. n = 5. (B) Quantification of the expression of *PiwiA* at 1, 3, 5, 7 and 10 dpa in the regenerated planarians after *DSP1* or control RNAi. (ns: no significant). (C) Whole-mount immunohistochemistry with antibodies against mitotic marker H3P^+^ in the regenerated planarians after DSP1 or control RNAi. Scale bars: 100 μm. (D) Quantification of the number of dividing cells in the regenerated planarians at 1, 2, 3 and 5 dpa after DSP1 or control RNAi. (ns: no significant). n = 5. **p < 0.01.

Figure S2 Co-knockdown of *DSP1* and *CnB* significantly accelerates the regeneration process in planarians.

Representative images of morphologic changes in the regenerated planarians at 7 and 10 dpa after Control RNAi, *DSP1* RNAi, *CnB* RNAi and *DSP1*+*CnB* RNAi. Scale bars: 250 μm. n = 20.
